# A case report of oculopharyngodistal myopathy with 126 CGG repeat expansions in *RILPL1*

**DOI:** 10.3389/fgene.2025.1472907

**Published:** 2025-02-27

**Authors:** Wenjing Wang, Tielun Yin, Xinyu Zhang, Zhaoxia Wang, Tianyun Wang, Shuo Zhang, Yingshuang Zhang, Dongsheng Fan

**Affiliations:** ^1^ Department of Neurology, Peking University Third Hospital, Beijing, China; ^2^ Beijing Key Laboratory of Biomarker and Translational Research in Neurodegenerative Diseases, Peking University Third Hospital, Beijing, China; ^3^ Key Laboratory for Neuroscience, National Health Commission/Ministry of Education, Peking University, Beijing, China; ^4^ Department of Neurology, Peking University First Hospital, Beijing, China; ^5^ Beijing Key Laboratory of Neurovascular Disease Discovery, Peking University First Hospital, Beijing, China; ^6^ Department of Medical Genetics, Center for Medical Genetics, Peking University Health Science Center, Beijing, China; ^7^ Key Laboratory for Neuroscience, Ministry of Education of China & National Health Commission of China, Neuroscience Research Institute, Peking University, Beijing, China

**Keywords:** oculopharyngodistal myopathy, RILPL1, CGG repeat, OPDM4, myopathy

## Abstract

**Background:**

Oculopharyngodistal myopathy (OPDM) is a rare hereditary muscle disease characterized by progressive ptosis, ophthalmoplegia, dysphagia, dysarthria, and distal muscle weakness. The genetic basis was identified in 2019 with CGG repeat expansions in the noncoding region of *LRP12*. Similar expansions in *GIPC1, NOTCH2NLC, and RILPL1* were later linked to OPDM, classifying the disease into OPDM1-4. OPDM4, associated with *RILPL1*, was discovered in 2022 with a few confirmed cases worldwide, leaving its clinical features and pathogenic mechanisms largely unexplored.

**Case presentation:**

We present a patient with OPDM4 who had suffered progressive ptosis, external ophthalmoplegia, pharyngeal weakness, facial muscle weakness, and distal limb weakness over the past 20 years. Electromyography (EMG) revealed myogenic damage and normal H-reflex latency. A biopsy of the left biceps brachii revealed myogenic changes with atypical rimmed vacuoles in some muscle fibers. Screening for extra-muscular system involvement revealed no obvious involvement of the heart or central nervous system. Genetic analysis confirmed 126 CGG repeat expansions in *RILPL1* and excluded abnormal CGG repeat expansions in *LRP12, GIPC1, and NOTCH2NLC*.

**Conclusion:**

This case broadens the spectrum of CGG repeat numbers in the *RILPL1* gene associated with OPDM4. In addition, systematic medical examinations revealed several new characteristics of OPDM4, which have not been reported previously, such as normal H reflex, potential mild cognitive impairment, etc. These findings expand our knowledge of the phenotypic spectrum of diseases caused by repeat CGG expansions in *RILPL1*.

## Introduction

Oculopharyngodistal myopathy (OPDM) is a rare hereditary muscle disease that is clinically defined by progressive symptoms including ptosis, ophthalmoplegia, dysphagia, dysarthria, and distal muscle weakness. Satoyoshi and Kinoshita first described OPDM in four Japanese families in 1977 (Satoyoshi and Kinoshita, 1977). Since then, more than 300 cases of OPDM across diverse ethnicities have been documented. Despite OPDM being a long-standing disease, its genetic basis remained unknown until recently. In 2019, CGG repeat expansions in the noncoding region of low-density lipoprotein receptor-related protein 12 (*LRP12*) were identified as causative for OPDM (Ishiura et al., 2019). Subsequently, similar expansions in the noncoding regions of GAIP C-terminus-interacting-protein 1 (*GIPC1*), NOTCH2 N-terminal-like protein C (*NOTCH2NLC*), and Rab interacting lysosomal protein like 1 (*RILPL1*) were subsequently linked to the OPDM phenotype (Deng et al., 2020; Ogasawara et al., 2020; Xi et al., 2021; Yu et al., 2021a; An et al., 2022; Yu et al., 2022; Zeng et al., 2022; Yang et al., 2023; YU et al., 2023; Eura et al., 2024; Ma et al., 2024). The OPDM subtypes associated with these genes can be classified into four types: OPDM1 to OPDM4. The four subtypes of OPDM have been reported to exhibit an autosomal dominant mode of inheritance (Ishiura et al., 2019; Deng et al., 2020; Ogasawara et al., 2020; Fukuda et al., 2021; Kumutpongpanich et al., 2021; Xi et al., 2021; Yu et al., 2021a; Yu et al., 2022; Zeng et al., 2022). Even though certain OPDM patients appear sporadic or autosomal recessive, pedigree analysis revealed that such individuals have asymptomatic fathers with extremely long CGG repeat expansions (>200 repeats) (Deng et al., 2020; Kumutpongpanich et al., 2021; Yu et al., 2021a). This phenomenon is likely attributable to a hypermethylation mechanism, which silences the transcription of the host genes in the expanded allele, whereas the normal allele compensates for the gene function, thereby producing an asymptomatic phenotype (Yu et al., 2021a). The causative genes for OPDM are still being discovered, such as *ABCD3* and *LOC642361/NUTM2B-AS1* (Cortese et al., 2024; Gu et al., 2024; Pongpakdee et al., 2024). Among these genes, the causative gene *RILPL1* for OPDM4 was only discovered very recently in 2022 (Yu et al., 2022). Currently, only approximately 20 patients are genetically confirmed to have OPDM4. These OPDM4 cases have been reported only in mainland China (Yu et al., 2022; Zeng et al., 2022; Yang et al., 2023; Eura et al., 2024). Given the rarity of reported OPDM4 cases, our understanding of its clinical features and pathogenic mechanisms remains highly limited. In this case report, we described a patient with a genetically confirmed diagnosis of OPDM4. This report aims to contribute to the limited body of literature on OPDM4, deepening our understanding of the characteristics of OPDM4.

## Case description

**FIGURE 1:**
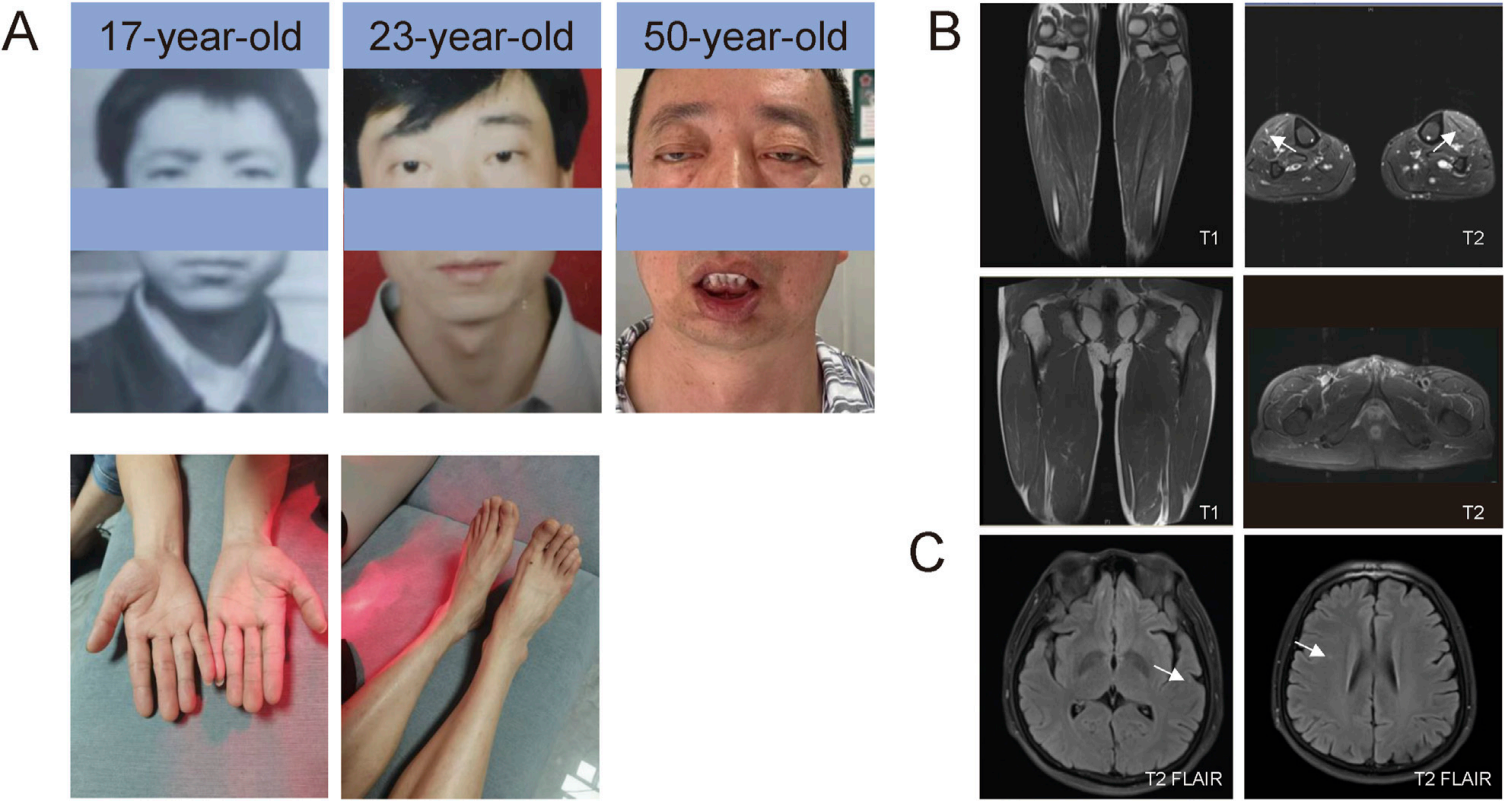
Photos of patients and MRI. **(A)** Facial photos at different ages and of the distal limbs. **(B)** T1- and T2-weighted MRI sequences of the legs. Muscle MRI showed T2 hyperintensities, suggesting edema of the lower legs (tibialis anterior). **(C)** T2/FLAIR sequences of the brain MRI. Brain MRI revealed T2/FLAIR hyperintensities, suggesting mild white matter demyelination.

A 50-year-old male was admitted to Peking University Third Hospital on 18 February 2024, due to progressive ptosis, pharyngeal weakness, and facial muscle weakness during the past 20 years. Initially, he experienced eye movement difficulties, accompanied by bilateral exophthalmos and incomplete eyelid closure. Five years ago, he developed voice changes, slow swallowing, and marked drooping of the lower lip (Figure 1; Table 1). He did not experience subjective limb weakness, sensory abnormalities, or other neurological symptoms. He was previously in good health, with no history of hypertension, diabetes, or coronary heart disease. He smoked half a pack of cigarettes and drank one bottle of beer daily for over 20 years but quit them 15 years ago. No other individuals in his family had similar symptoms. Neurological examination revealed dysarthria, external ophthalmoplegia, facial muscle weakness and pharyngeal weakness (Figure 1A). Muscle strength was Grade IV for distal muscles of upper limbs, Grade IV+ for distal muscles of lower limbs, and Grade V for proximal muscles of both limbs. Tendon reflexes were absent with negative pathological signs in both limbs (Table 1).

**TABLE 1:** Summary of clinical manifestations.

Clinical manifestations	Our OPDM4 case	Previous OPDM4 cases (Kumutpongpanich and Liewluck, 2022; Yu et al., 2022; Zeng et al., 2022)
Sex (male/female)	Male	Predominantly male
Age of onset (years)	30	22.2 ± 7.0
Initial symptoms	Ptosis; External ophthalmoplegia	ocular > limb
Pharyngeal weakness	+	94% (15/16)
Facial muscle weakness	+	94% (15/16)
Distal limb weakness	+	75% (12/16)
Loss of ambulation	−	17% (1/6)
Tendon reflex	−	−
MoCA score	26	NA
MMSE score	25	NA
Cardiomyopathy	− (LVEF 71%)	−
Electrocardiogram	Sinus arrhythmia	NA
Respiratory insufficiency	—	NA
CNS involvement	Mild white matter demyelination	−
Digestive system involvement	Splenomegaly	NA
Serum CK (IU/L)	110 IU/L (normal limits: 25–192 IU/L)	375.20 ± 191.94 IU/L (normal limits: 70–170 IU/L)
Muscle MRI	Edema of the lower legs	Atrophy and fibro-fatty infiltration in some cases
Rimmed vacuoles (RVs)	+	+
Gene with CGG expansion	*RILPL1*	*RILPL1*
Pathological repeat numbers	126	139–197

MoCA, Montreal Cognitive Assessment; MMSE, Mini-Mental State Examination; CK, Creatine kinase; CNS, central nervous system; LVEF, Left Ventricular Ejection Fraction; NA, not available.

**FIGURE 2:**
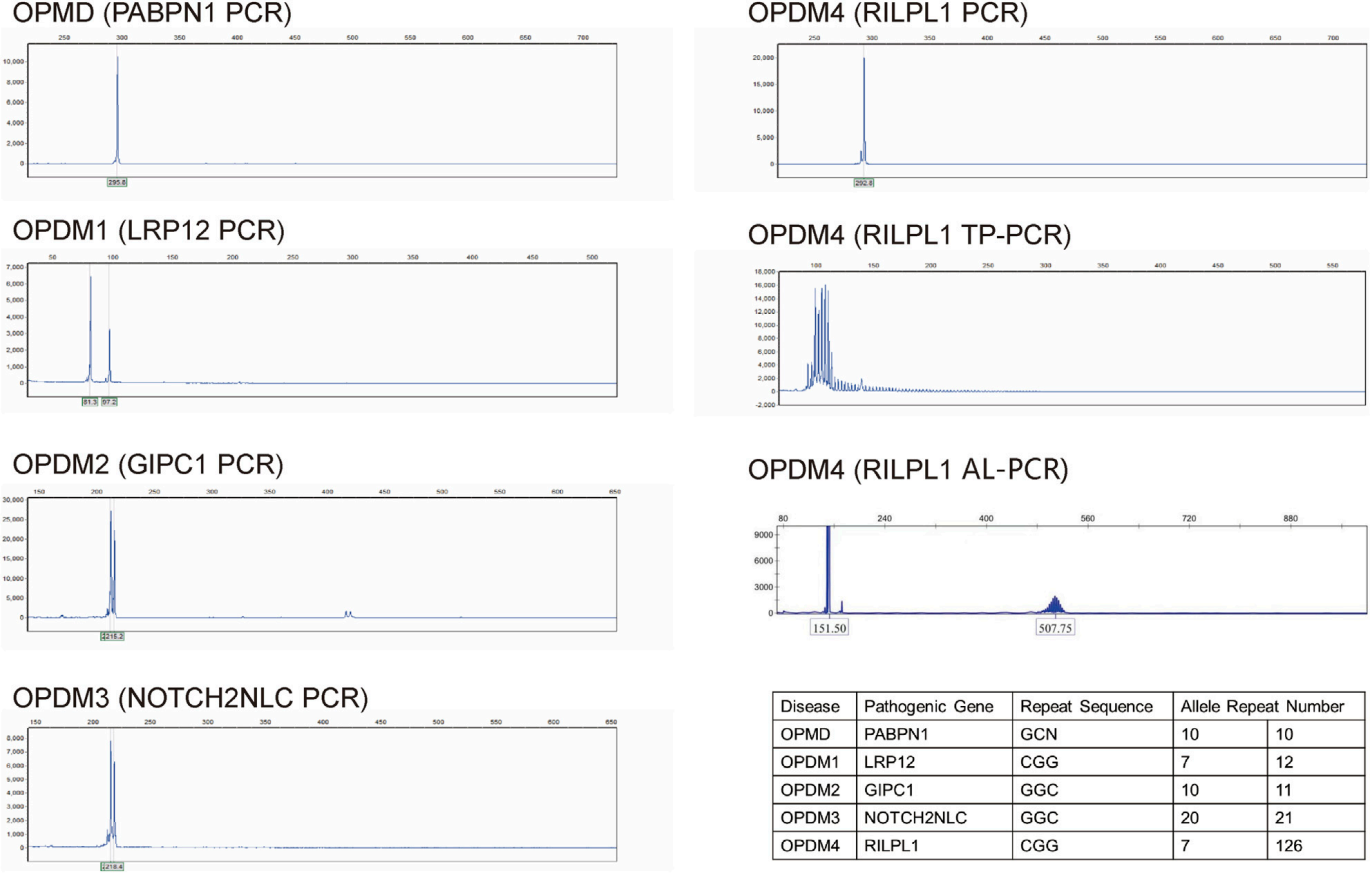
Dynamic mutation analysis of the four causative genes for OPDM (*LRP12*, *GIPC1*, *NOTCH2NLC*, and *RILPL1*) and OPMD (*PABPN1*) showed that repeat numbers in the *LRP12, GIPC1, NOTCH2NLC* and *PABPN1* loci were within the normal range. Triplet repeat-primed PCR (TP-PCR) revealed that the patient had CGG repeat numbers of 7 and >78 in the OPDM4 causative gene *RILPL1*, with one allele exceeding the normal range. Fluorescence amplicon length analysis PCR (AL-PCR) technology detected 126 CGG repeats in *RILPL1*. Repeat numbers in the *LRP12*, *GIPC1*, *NOTCH2NLC*, and *PABPN1* loci were within normal ranges.

Comprehensive auxiliary examinations were performed. Laboratory tests, including Routine blood, urine, and stool tests, along with biochemical tests, coagulation function, thyroid function, and autoantibodies, were all within normal ranges, and creatine kinase was 110 IU/L (normal limits: 25–192 IU/L). Electrophysiological examinations showed normal motor and sensory nerve conduction in all four limbs, with normal F-wave latency and occurrence rates of 50%–100% in both the median and ulnar nerves, and normal H-reflex latency in both tibial nerves. Electromyography (EMG) revealed myotonic discharges and myogenic damage in the left first interosseous muscle, right gastrocnemius, left biceps brachii, and left quadriceps. Muscle MRI revealed mild muscle edema in the lower legs (Figure 1B), and brain MRI revealed mild white matter demyelination and bilateral mastoiditis (Figure 1C). A biopsy of the left biceps brachii revealed myogenic changes. The muscle fibers exhibited mild size variability, with a few small round atrophic and degenerative fibers, and individual muscle fibers containing vacuoles. We also conducted cognitive function tests on the patient who had a middle school education level. The patient scored 26 points on the Montreal Cognitive Assessment (MoCA) and 25 points on the Mini-Mental State Examination (MMSE). An electrocardiogram revealed sinus arrhythmia. Echocardiography revealed no significant abnormalities. Color Doppler ultrasound of the digestive system indicated splenomegaly (Tables 1, 2). Genetic testing, including dynamic mutation analysis of the four causative genes for OPDM (*LRP12*, *GIPC1*, *NOTCH2NLC*, and *RILPL1*) revealed that the patient had CGG repeat numbers of 7 and >78 in the OPDM4 causative gene *RILPL1*, with one allele exceeding the normal range. Fluorescence amplicon length analysis PCR (AL-PCR) technology revealed that *RILPL1* contains 126 CGG repeats, in which the primers *RILPL1*-F (5′-Fam-CCATTGAGCGCAACTCGGATC-3′) and *RILPL1*-AL-R (5′-GGG​TGT​GCG​GGC​CCG​G-3′) were used. Repeat numbers in the *LRP12*, *GIPC1*, and *NOTCH2NLC* loci were within the normal range. Additionally, testing for dynamic mutations in the poly-adenine-binding protein nuclear 1 (*PABPN1*) gene, which is associated with oculopharyngeal muscular dystrophy (OPMD) (Malerba et al., 2017), revealed normal GCN repeat numbers. Whole exome sequencing and mitochondrial gene testing did not reveal any pathogenic or likely pathogenic variants explaining the patient’s phenotype (Figure 2).

**TABLE 2:** Summary of electrophysiological data.

CMAP amplitude (mV)	Left median nerve	4.4
	Right median nerve	4.9
	Left ulnar nerve	5.7
	Right ulnar nerve	5.3
	Left peroneal nerve	3.9
	Right peroneal nerve	4.6
	Left tibial nerve	5.5
	Right tibial nerve	5.2
F-wave frequency (%)	Left medianus	75
	Right medianus	50
	Left ulnaris	95
	Right ulnaris	100
H-reflex latency (ms)	Left tibialis	28.7
	Right tibialis	29.4
EMG spontaneous activity	Right biceps brachii	Fib −; PSW −
	Right gastrocnemius caput mediale	Fib 2+∼3+; PSW 2+∼3+; atypical myotonic discharges
	left first dorsal interosseous muscle	Fib 3+; PSW 3+; atypical myotonic discharges
	left vastus medialis muscle	Fib −; PSW −
EMG Voluntary motor unit potentials	Right biceps brachii	Amp 573 μV; Dur 9.9 ms↓
	Right gastrocnemius caput mediale	Amp 682 μV; Dur 8.4 ms↓
	left first dorsal interosseous muscle	Amp 667 μV; Dur 6.6 ms↓
	left vastus medialis muscle	Amp 506 μV; Dur 9.3 ms↓

Fib, fibrillation potential; PSW, Positive sharp wave; Amp, Amplitude; Dur, Duration.

## Discussion

OPDM4 is an exceptionally rare disorder (Yu et al., 2022; Zeng et al., 2022; Yang et al., 2023; Eura et al., 2024). It is urgently needed to further accumulate cases to deepen our understanding of the clinical features and pathogenic mechanisms of OPDM4. This case report presents comprehensive clinical manifestations, diagnostic approaches, and genetic findings, with the goal of enhancing and expanding our knowledge of this rare disorder. We found that the CGG repeat count in the RILPL1 gene was 126, which was smaller than the previously reported minimum pathogenic repeat count of 139. Through systematic medical examinations, we also discovered some new characteristics of OPDM4, which have not been reported previously, such as a normal H reflex, and potential mild cognitive impairment.

In our case, we broadened the spectrum of CGG repeat numbers in the *RILPL1* gene associated with OPDM4. Among a large Chinese cohort, 11 out of 51 OPDM patients (21.6%) were diagnosed with OPDM4. The average age of onset was approximately 24 years, with repeat sizes ranging from 139 to 197 (Yu et al., 2022). In this case, the patient had 126 CGG repeat expansions in *RILPL1*, which was less than 139. Therefore, it is reasonable to deduce that CGG repeat expansions ranging from 126 to 197 are pathological.

With the progress of research, OPDM1-3 have been found to affect the extramuscular system. Some OPDM1 patients have been reported to develop respiratory insufficiency, cardiomyopathy of undetermined cause (atrial fibrillation, heart failure, second-degree atrioventricular block, and dilated cardiomyopathy), and neurologic abnormalities (dementia, idiopathic Parkinson disease and mild cognitive impairment) (Saito et al., 2020; Kumutpongpanich et al., 2021; Shimizu et al., 2022; Hobara et al., 2025). GGC repeat expansions in OPDM2 causative gene *GIPC1* have been found to be associated with movement disorders (Fan et al., 2022; Pan et al., 2022; Zhou et al., 2022; Murayama et al., 2024). OPDM3 and neuronal intranuclear inclusion disease (NIID) share the same cause, GGC repeat expansions in *NOTCH2NLC*. The pathological process of OPDM3 extends beyond the oculopharyngeal muscles, and involves various extraskeletal muscle organs, including the central and peripheral nervous systems, as well as cardiac, respiratory, and gastrointestinal systems (Ishiura et al., 2019; Tian et al., 2019; Ogasawara et al., 2020; Cao et al., 2021; Yu et al., 2021a; Yu et al., 2021b; Boivin and Charlet-Berguerand, 2022; Huang et al., 2022; Ma et al., 2024). However, there is currently insufficient research on extramuscular system involvement in OPDM4 patients. Therefore, screening for extramuscular system involvement is also crucial for OPDM4 patients, as muscular symptoms may be only a part of the phenotypic spectrum caused by CGG repeat expansions in *RILPL1*. In this case, we thoroughly screened for extramuscular system involvement, such as the central nervous system, heart, and digestive system. In this patient, brain MRI revealed mild white matter demyelination. He scored 26 points on the MoCA and 25 points on the MMSE, which indicates potential mild cognitive impairment. Besides a brief history of smoking and alcohol use, he has no vascular risk factors such as hypertension or diabetes. There is a possibility that the mild white matter demyelination and potential mild cognitive impairment were related to OPDM4. Echocardiography and EEG showed no evidence of abnormalities in heart structure or cardiac functions. He had no breathing difficulty until now. Doppler ultrasound of the digestive system indicated splenomegaly, but it is unclear whether this is related to the disease. These findings expand our knowledge of the phenotypic spectrum of OPDM4 caused by repeat CGG expansions in *RILPL1*.

We conducted comprehensive electrophysiological examinations on this patient, which provided insights into the affected muscle sub-areas. Notably, the patient’s H-reflex was normal, despite the complete absence of tendon reflexes. This discrepancy is intriguing because the reflex arcs for the H-reflex and tendon reflexes are similar, and involve the afferent pathway, central processing, efferent pathway, and effector. The key distinction lies in the fact that the H-reflex is triggered by electrical stimulation, whereas the tendon reflex is elicited by mechanical stimulation, such as tapping the tendon. The H-reflex primarily involves type Ia sensory fibers activated by electrical stimulation of sensory nerves, whereas the tendon reflex is triggered by muscle stretch, stimulating sensory nerves within muscle spindles. Given that the patient’s nerve conduction was normal, it is reasonable to infer that the affected muscle sub-area in the patient was muscle spindles. This finding suggested that muscle spindles could be susceptible or affected early in OPDM4 patients.

Our case also provides valuable evidence for differentiating OPDM4 from OPMD and other OPDM subtypes. OPDM and OPMD are similar and even believed to be indistinguishable in terms of their myopathological features. However, OPMD is caused by an alanine expansion mutation in *PABPN1* gene (Brais et al., 1998). Clinically, OPMD is characterized by oculopharyngeal muscle involvement and rimmed vacuolar pathology, with proximal limb muscle weakness being a typical feature, rather than distal weakness (Victor et al., 1962; Tomé et al., 1997). Our patient exhibited distal limb weakness, and genetic testing revealed no abnormalities in the *PABPN1* gene. In addition, this case confirms the differences in clinical features between OPDM4 and OPDM1-3, consistent with previous studies. It has been reported that 90% (9/10) of OPDM4 patients initially present with ptosis or dysarthria, and 60% do not exhibit distal muscle weakness until 10–20 years after these initial symptoms (Yu et al., 2022). On the contrary, distal-limb weakness was the most common initial symptom in OPDM1 (3 out of 4, 75%), OPDM2 (14 out of 19, 73.68%), and OPDM3 (8 out of 9, 87.5%) (Deng et al., 2020; Ogasawara et al., 2020; Xi et al., 2021; Yu et al., 2021a; Yu et al. 2021b; Yu et al. 2022; Zeng et al., 2022; Yang et al., 2023; Eura et al., 2024; Ma et al., 2024). In this case, 20 years ago, the patient experienced initial symptoms of ptosis and external ophthalmoplegia. To date, the patient has not reported subjective limb weakness, although neurological examination revealed a mild decrease in distal muscle strength. His clinical presentation was consistent with the distinct characteristics previously reported.

All OPDM subtypes exhibit autosomal dominant inheritance (Ishiura et al., 2019; Deng et al., 2020; Ogasawara et al., 2020; Kumutpongpanich et al., 2021; Xi et al., 2021; Yu et al., 2021a; Yu et al., 2022). Interestingly, this patient had no relevant family history. Due to the rarity of reported OPDM4 cases, it was difficult to deduce the exact mode of inheritance. However, some findings have been reported for other OPDM subtypes. In OPDM1-3, the CGG repeat has been observed to expand or contract over generations in some families. Currently, asymptomatic carriers with large expansions (>200–300) are reported to be fathers of affected offspring with shorter expansions, indicating a bias towards the male germline (Deng et al., 2020; Fukuda et al., 2021; Kumutpongpanich et al., 2021; Yu et al., 2021a). Similar repeat contractions in affected offspring have been reported in asymptomatic parents, typically fathers, with fragile X messenger ribonucleoprotein 1 (FMR1) CGG repeats in Fragile X syndrome (Nolin et al., 2019). One possible explanation is that an expansion beyond a certain length could lead to hypermethylation in the promoter region, thus silencing gene transcription and preventing the production of toxic mRNA (Kumutpongpanich et al., 2021). In our case, the patient’s mother is currently 81 years of age and in very good health, without any symptoms similar to those of this patient. His father passed away at the age of 73 years because of myocardial infarction. The father did not exhibit clinical manifestations similar to those of the patient throughout life. Based on the patterns observed in other OPDM subtypes, we speculate that the CGG repeat in *RILPL1* was inherited from the patient’s asymptomatic father.

Our study has several limitations. Although muscle pathology revealed rimmed vacuoles (RVs), a hallmark of OPDM, we were unable to detect another key marker, p62-positive nuclear inclusions. This limitation arose because the biopsy was performed years ago at another hospital, and the patient declined a repeat procedure. Furthermore, conducting genetic testing on the patient’s mother would strengthen our hypothesis that the CGG repeat expansion in *RILPL1* was inherited from his asymptomatic father, but consent was not obtained.

In conclusion, this case broadens the genetic and phenotypic spectrum of CGG repeat numbers in the *RILPL1* gene associated with OPDM4. In addition, systematic medical examinations revealed some new characteristics of OPDM4. For OPDM4, we still need to accumulate a large number of cases to summarize its clinical features and causative mechanisms. With more OPDM4 cases and muscle-related examinations reported, enhancing our understanding of the relationship between genotype and phenotype is meaningful. In terms of physical examination and auxiliary tests, we should not limit ourselves to muscle-related examinations, which will enhance our understanding of the phenotypic spectrum caused by CGG repeat expansions in *RILPL1*.

## Data Availability

The original contributions presented in the study are included in the article/supplementary material, further inquiries can be directed to the corresponding author.
